# Qi-Dong-Huo-Xue-Yin balances the immune microenvironment to protect against LPS induced acute lung injury

**DOI:** 10.3389/fphar.2023.1200058

**Published:** 2023-05-24

**Authors:** Tian zhao, Le Wang, Yongjun Zhang, Wu Ye, Juan Liu, Haiyan Wu, Fei Wang, Tingyu Tang, Zhijun Li

**Affiliations:** ^1^ Department of Respiratory Medicine, Zhejiang Hospital, Hangzhou, Zhejiang, China; ^2^ The Second Clinical Medical College Affiliated to Zhejiang University of Traditional Chinese Medicine, Hangzhou, Zhejiang, China; ^3^ The Cancer Hospital of the University of Chinese Academy of Sciences Zhejiang Cancer Hospital, Hangzhou, Zhejiang, China

**Keywords:** Qi-Dong-Huo-Xue-Yin, traditional Chinese medicine, Tregs, macrophage, Foxp3, ALI

## Abstract

COVID-19 induces acute lung injury (ALI)/acute respiratory distress syndrome (ARDS) and leads to severe immunological changes that threatens the lives of COVID-19 victims. Studies have shown that both the regulatory T cells and macrophages were deranged in COVID-19-induced ALI. Herbal drugs have long been utilized to adjust the immune microenvironment in ALI. However, the underlying mechanisms of herbal drug mediated ALI protection are largely unknown. This study aims to understand the cellular mechanism of a traditional Chinese medicine, Qi-Dong-Huo-Xue-Yin (QD), in protecting against LPS induced acute lung injury in mouse models. Our data showed that QD intrinsically promotes Foxp3 transcription via promoting acetylation of the Foxp3 promoter in CD4^+^ T cells and consequently facilitates CD4^+^CD25^+^Foxp3^+^ Tregs development. Extrinsically, QD stabilized β-catenin in macrophages to expedite CD4^+^CD25^+^Foxp3^+^ Tregs development and modulated peripheral blood cytokines. Taken together, our results illustrate that QD promotes CD4^+^CD25^+^Foxp3^+^ Tregs development via intrinsic and extrinsic pathways and balanced cytokines within the lungs to protect against LPS induced ALI. This study suggests a potential application of QD in ALI related diseases.

## Introduction

Infection of SARS-CoV-2 results in hyperactivation of the immune system and persistent inflammation causes acute lung injury (ALI) (Grasselli et al., 2020; Rendeiro et al., 2021). Mice infected with SARS-CoV-2 develop a loss of pulmonary function with pathological features of ALI (Leist et al., 2020). ALI leads to high mortality since accumulated inflammatory cells and cytokines damage the capillary endothelial and alveolar epithelial barrier (Bellani et al., 2016). The current strategy to treat ALI is to inhibit hyperactivated inflammation, although no effective pharmacological therapy is yet available (Bos and Ware, 2022; Ning et al., 2023). Hence, it is of importance to understand how inflammation is over-activated in ALI and identify effective drugs that prohibit the hyperactivated inflammation.

Immune cell populations balance between damage and repair after tissue injury and macrophages have predominant roles in protecting the host against injury (Bellani et al., 2016; Quinton et al., 2018). Macrophages are involved in every stage of ALI (Chen et al., 2020a). In the exudative phase of ALI, local macrophages shift to the M1 phenotype and mediate the release of inflammatory cytokines (Laskin et al., 2019), and persistent accumulation of these proinflammatory factors finally damage the lung tissues (Zhuo et al., 2019). In the rehabilitation phase of ALI, however, macrophages shift from the M1 to the M2 phenotype and mediate the release of anti-inflammatory factors such as IL-10 and TGF-β to control inflammation (Herold et al., 2011). Besides macrophages, CD4^+^CD25^+^Foxp3^+^ Tregs (regulatory T cells) play a critical role in resolving acute lung injury (D'Alessio et al., 2009). Increased recruitment of CD4^+^CD25^+^Foxp3^+^ Tregs or increased differentiation of CD4^+^CD25^+^Foxp3^+^ Tregs provide anti-inflammatory effects in ALI models (Wang et al., 2012; Xie et al., 2021). Tregs balance the immune environment via elevating the alveolar cytokine TGF-β (D'Alessio et al., 2009). Additionally, Tregs are required for lung epithelial proliferation, which promotes lung injury recovery (Mock et al., 2014). Based on these findings, sustaining a balanced immune environment, especially the portions of macrophages and Tregs and providing a suitable level of TGF-β, would largely benefit the host from acute lung injury.

Multiple herbal drugs have been used to fight against the COVID-19 pandemic, and one of the major aims of using herbal drugs is to inhibit inflammation (Jan et al., 2021). Among them, several traditional Chinese medicine (TCM) formulas have been proven beneficial in controlling excessive inflammation and preventing ALI caused by SARS-CoV-2 infection (Li et al., 2021). Various attempts have been made with TCM decoctions to protect against ALI (Ding et al., 2020; Guo et al., 2021). A classical TCM decoction, Xuanfei Baidu, effectively reduces ALI by regulating immune cell infiltration (Wang et al., 2022), while Liang-Ge-San attenuates ALI through decreasing the release of IL-6 from macrophages (Yang et al., 2019). Interestingly, a recent report showed that Gegen Qinlian decoction attenuated ALI via regulating intestinal microbiota (Li et al., 2022). Qi-Dong-Huo-Xue-Yin (QD) is a TCM that consists of *Astragalus membranaceus*, *Radix Ophiopogonis*, *Polygonum cuspidatum*, *Angelica sinensis*, and *Rheum officinale*. Study has shown that QD inhibits ALI by regulating the levels of cytokines in bronchoalveolar lavage fluid (Xu et al., 2018). Moreover, it has been shown that QD facilities the expression of ENaC-α and AQP-1 and ameliorates pulmonary edema in acute lung injury mice (Ma et al., 2020). However, cellular and molecular mechanisms underlying QD mediated inflammation inhibition are largely unknown.

Here, we first analyzed the portion of Tregs in a lipopolysaccharide-induced ALI mouse model and revealed an epigenetic regulation of Foxp3 expression in the presence of QD. In addition to the intrinsic regulatory network, we found that QD also functioned on macrophages by stabilizing the protein level of β-Catenin, which further promoted the development of CD4^+^CD25^+^Foxp3^+^ Tregs and balanced the cytokines in the lung to protect against ALI.

## Materials and methods

### Preparation and utilization of QD decoction

QD Decoction was prepared as previously described (Akimova et al., 2010). Briefly, a mixture of 20 g *A. membranaceus*, 12 g *Radix Ophiopogonis*, 20 g *P. cuspidatum*, 12 g *A. sinensis*, and 9 g *R. officinale* were boiled in 700 mL water for 30 min and steeped for 30 min. The decoction was subsequently concentrated for 40 min and diluted to concentration of 20 g/mL. The working concentration of QD for cell culture is 0.2 g/mL and 1 g QD per 10 g mouse weight.

### Animals

Wildtype (control) mice used in this study refer to specific-pathogen-free BALB/c mice. Myeloid β-Catenin deficient mice were generated by mating β-catenin^flox^ mice (The Jackson Laboratory, Bar Harbor, ME) and Lysozyme M^cre^ (The Jackson Laboratory, Bar Harbor, ME).

All animals were housed and maintained in specific pathogen-free conditions according to the recommendation of Guide for the Care and Use of Laboratory Animals of the National Institutes of Health. Animal experiments were approved by the institutional biomedical research ethics committee of Zhejiang Hospital (Zhejiang, China).

### Establishment of ALI model

ALI models were established as previously described (D'Alessio et al., 2009). Briefly, 100 μg/mouse LPS (*Escherichia coli* 055:B5, Sigma-Aldrich) was instilled via the catheter inserted into the lumen of trachea from the neck. The sham group received 100 μg of 0.9% NaCl instead of LPS. All ALI mice were sacrificed 24 h post-surgery. Lung tissues, bronchoalveolar lavage fluid, and peripheral blood were collected.

### Histopathology

Lungs were fixed in 4% paraformaldehyde for 24 h and embedded in paraffin. Sections of 5 μm were prepared for H&E staining according to standard procedure. The lung injury score was evaluated as previously described (D'Alessio et al., 2009).

### Permeability index analysis

Permeability index analysis was performed as previously described (Zhou et al., 2019). Briefly, human serum albumin (i.v. 25 μg; Signa-Aldrich, MO) was administrated 1 h prior to sacrificing the animal. ELISAs were performed to determine the level of human albumin concentration and pulmonary permeability index was defined as the human albumin concentration in BALF/serum ratio.

### Myeloperoxidase activity analysis

Myeloperoxidase (MPO) activity measurements were performed as previously described (McCabe et al., 2001). The lungs were homogenized and centrifuged. Supernatants were collected and analyzed for MPO activity by spectrophotometry.

### Cell isolation, culture, and treatment

The peripheral blood or spleen T cells were purified using the EasySepTM mouse T cell isolation kit (STEMCELL Technologies, Vancouver, BC, Canada) according to the manufacturer’s instructions. T cells were cultured in Advanced RPMI 1640 (Invitrogen, 12,633) supplemented with 10% FBS (Invitrogen), penicillin–streptomycin (Invitrogen), 55 μM β-mercaptoethanol, and 2 mM glutamine. Peritoneal macrophages were lavaged from mice 4 days after injection of 3 mL sterile thioglycollate (Sigma-Aldrich). Cells were cultured in RPMI 1640 with 10% heat-inactivated FBS (Invitrogen) plus 1% penicillin-streptomycin (Invitrogen) and 1% glutamine (Invitrogen). RAW264.7 cells were cultured in DMEM/high glucose supplemented with 10% heat-inactivated FBS and penicillin–streptomycin (Invitrogen). siRNAs (ordered from Ribo bio, Guangzhou) were transfected using Lipofectamine RNAiMax reagent (Invitrogen) and incubated with macrophages for 24 h. For QD pre-treatment, 0.2 g/mL QD was added to macrophage culture medium and cells were incubated for 3 h. After sorted T cells were stimulated with LPS (1 μg/mL) for 6 h, macrophages and T cells were co-cultured at ratio of 1:10 as described previously (Zhu et al., 2017). Cycloheximide was used at the concentration of 50 mg/mL just before the cells were analyzed.

### Flow cytometry

FITC-conjugated anti-CD4 and PE-conjugated anti-CD25 and respective isotype antibodies (553,729 and 558,642, BD Biosciences) were used for FACS analysis of Tregs. To stain Foxp3, mouse Foxp3 buffer set (560,409, BD Biosciences) was utilized. Lymphocytes were gated with characteristic low forward scatter/side scatter, using a FACS Aria instrument and FACS Diva for data acquisition (Becton Dickinson).

### ELISAs

ELISAs were performed according to the manufacturers’ protocols. The following kits were utilized in this study: Mouse IL-17A ELISA Kit (ab199081, Shanghai), Mouse TGF beta 1 ELISA Kit (ab119557, Shanghai), Mouse IL-23 ELISA Kit (ab119545, Shanghai), Mouse TNF alpha ELISA Kit (ab208348, Shanghai), and Human serum albumin ELISA kit (Cayman Chemical, Ann Arbor, MI).

### Western blot and chromatin immunoprecipitation

Cells were lysed in RIPA lysis buffer and Western blots were performed according to standard procedures. The antibodies used in this study are as follows: Anti-Histone H3 antibody - Nuclear Marker and ChIP Grade (ab1791, abcam), Anti-Histone H3 (acetyl K9) antibody [Y28] - ChIP Grade (ab32129, abcam), Anti-HDAC1 antibody [EPR460 (2)] (ab109411, abcam), Anti-HDAC2 antibody [Y461] (ab32117, abcam), Anti-HDAC3 antibody [Y415] (ab32369, abcam), Anti-HDAC4 antibody [EPR22937-157] (ab235583, abcam), Anti-HDAC6 antibody [EPR1698 (2)] (ab133493, abcam), Anti-HDAC9 antibody [EPR5223] (ab109446, abcam), Anti-beta Actin antibody [mAbcam 8226] - Loading Control (ab8226, abcam), Anti-PTEN antibody [EPR22636-122] (ab267787, abcam), Anti-beta Catenin antibody [E247] - ChIP Grade (ab32572, abcam), Anti-beta Catenin non-phospho (active) S37/T41 antibody [EPR23969-131] (ab246504, abcam), Anti-GSK3 beta antibody [Y174] (ab32391, abcam), and Anti-GSK3 beta (phospho S9) antibody (ab107166, abcam). The chromatin immunoprecipitation assay (ChIP) was performed with the Pierce Magnetic ChIP kit (26,157, Thermo) according to manufacturer’s instructions using Anti-Histone H3 antibody - Nuclear Marker and ChIP Grade (ab1791, abcam) and Anti-Histone H3 (acetyl K9) antibody [Y28] - ChIP Grade (ab32129, abcam).

### Colorimetric HDAC activity assay

HDAC activity was analyzed in cell lysates using a colorimetric HDAC activity kit (BioVision).

### Quantitative PCR (qPCR)

Total RNA was extracted using the Total RNA Extraction Reagent (R401-01, Vazyme), then reverse-transcribed to cDNA using the HiScript III first Strand cDNA Synthesis Kit + gDNA wiper (R312-01, Vazyme). qPCR was performed on the CFX96 Touch™ Real-Time PCR Detection System (Bio-Rad) using AceQ universal SYBR qPCR Master Mix (R511-01, Vazyme). Primers were listed as follow.

**Table:** 

GAPDH	Forward 5′-ACC ACA GTC CAT GCC ATC AC-3′
	Reverse 5′-TCC ACC ACC CTG TTG CTG TA-3′
Foxp3	Forward 5′-GCA​CAT​TCC​CAG​AGT​TCC​TCC-3′
	Reverse 5′-CCT​ATC​ATC​CCT​GCC​CCC​A-3′
HDAC1	Forward 5′- TGG​CCA​TCC​TGG​AAG​ACG​A-3′
	Reverse 5′- CAT​GAC​CGG​CTT​GAA​CCA​CC-3′
HDAC2	Forward 5′-CAG​TTG​CCC​TTG​ATT​GTG​AGA​TTC-3′
	Reverse 5′-GCT​ATC​CGC​TTG​TCT​GAT​GCT​C-3′
HDAC3	Forward 5′-ACG​TGG​GCA​ACT​TCC​ACT​AC-3′
	Reverse 5′-GAC​TCT​TGG​TGA​AGC​CTT​GC-3′
HDAC4	Forward5′-CACACACTCCTCTACGGCACA AAT-3′
	Reverse5′-ACCTTGAAG ACC​AGC​TCC​ACT​ACA​CA-3′
HDAC6	Forward 5′-AAG​GCC​ACT​GGG​AGG​CCA​CT-3′
	Reverse 5′-TGT​GCG​CAG​GCA​AAG​GTG​CT-3′
HDAC9	Forward 5′-GCC​TTT​TAG​GTG​TCC​CTA​TT-3′
	Reverse 5′-ACA​TCC​ATG​GAC​TAG​ACA​GC-3′
IL-17A	Forward 5′-GAA​ACT​CAT​CGT​GAA​GTC​AAA​CAT​TTA​A-3′
	Reverse 5′-AGA​TTT​TCT​ATA​GCT​CTT​TCT​TCC​AGT​G-3′
TGF-β	Forward 5′-AAC​AAT​TCC​TGG​CGT​ACC​TT-3′
	Reverse 5′-TCC​TTC​CAC​AGT​ATG​CTC​GTA-3′
ROR-γt	Forward 5′-TCC​ATA​TTT​GAC​TTT​TCC​CAC​T-3′
	Reverse 5′-GAT​GTT​CCA​CTC​TCC​TCT​TCT​C-3′
TNF-α	Forward 5′-TCT​ACT​GAA​CTT​CGG​GGT​GAT-3′
	Reverse 5′-ACT​TGG​TGG​TTT​GTG​AGT​GTG​A-3′
IL-1β	Forward 5′-TAA​TGA​AAG​ACG​GCA​CAC​CCA​C-3′
	Reverse 5′-GCT​CTG​CTT​GTG​AGG​TGC​TGA-3′
	Forward 5′-CAA​TCT​ACT​AAT​GCT​AAT​ACT​GTT​TCG-3′
	Reverse 5′-CCT​CAG​GAT​TGC​CTT​TAC​CA-3′

### Statistical analysis

All results were repeated independently at least three times and are presented as the mean ± SEM. Two-tailed unpaired Student’s t-test (for comparison of two groups) or one-way ANOVA followed by Tukey’s *post hoc* test (for multi-group comparison) was performed using GraphPad Prism 9. A *p*-value <0.05 was considered statistically significant.

## Results

### QD alleviates acute lung injury via boosting T cell development

**FIGURE 1:**
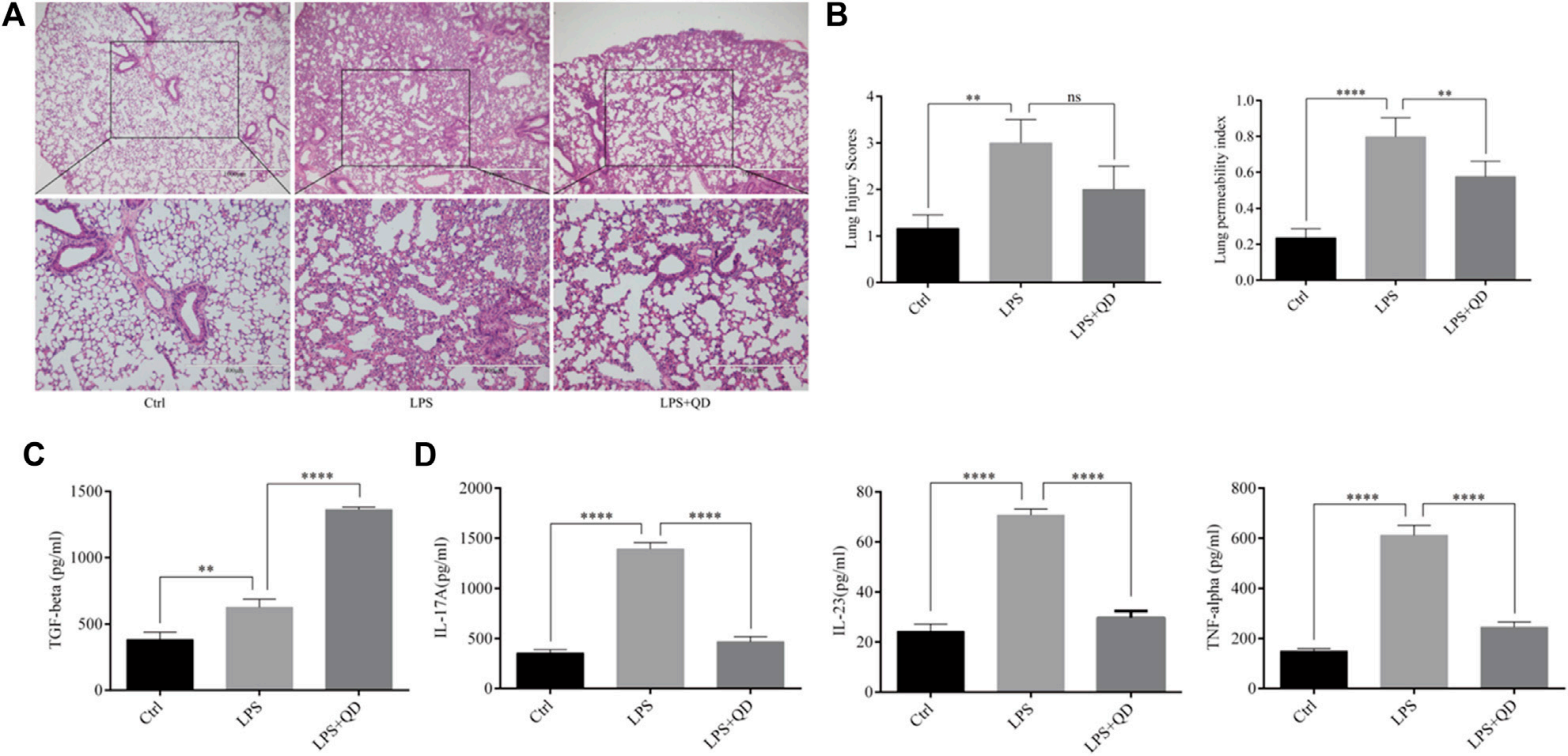
QD attenuates LPS induced ALI **(A)** H&E staining of lung tissues from control and ALI model mice treated with or without QD (×40 magnification). Ctrl stands for control. **(B)**. Histopathological mean lung injury scores and pulmonary permeability index calculated from six control and ALI model mice treated with or without QD. **(C)**. TGF-β level in BALF of control and ALI model mice treated with or without QD. **(D)**. IL-17A, IL-23, and TNF-α levels in BALF of control and ALI model mice treated with or without QD.

To understand the effect QD has on acute lung injury, we first induced acute lung injury with LPS. QD treatment significantly attenuated the lung injury as revealed by reduced inflammatory cell infiltration and the alveolar wall thickness (Figure 1A). Additionally, both lung injury scores and the lung permeability index (LPI) were significantly decreased in QD treatment group in comparison to the LPS treatment alone group (Figure 1B), suggesting that QD treatment alleviated acute lung injury induced by LPS. We then analyzed the inflammatory factors in bronchoalveolar lavage fluid (BALF) and found that TGF-β was increased after QD treatment (Figure 1C), while pro-inflammatory factors IL-17A, IL-23, and TNF-α were significantly decreased after QD treatment (Figure 1D). These data suggested that QD treatment effectively inhibited pro-inflammatory cytokines in acute lung injury yet induced TGF-β, a known factor that alleviate acute lung injury.

**FIGURE 2:**
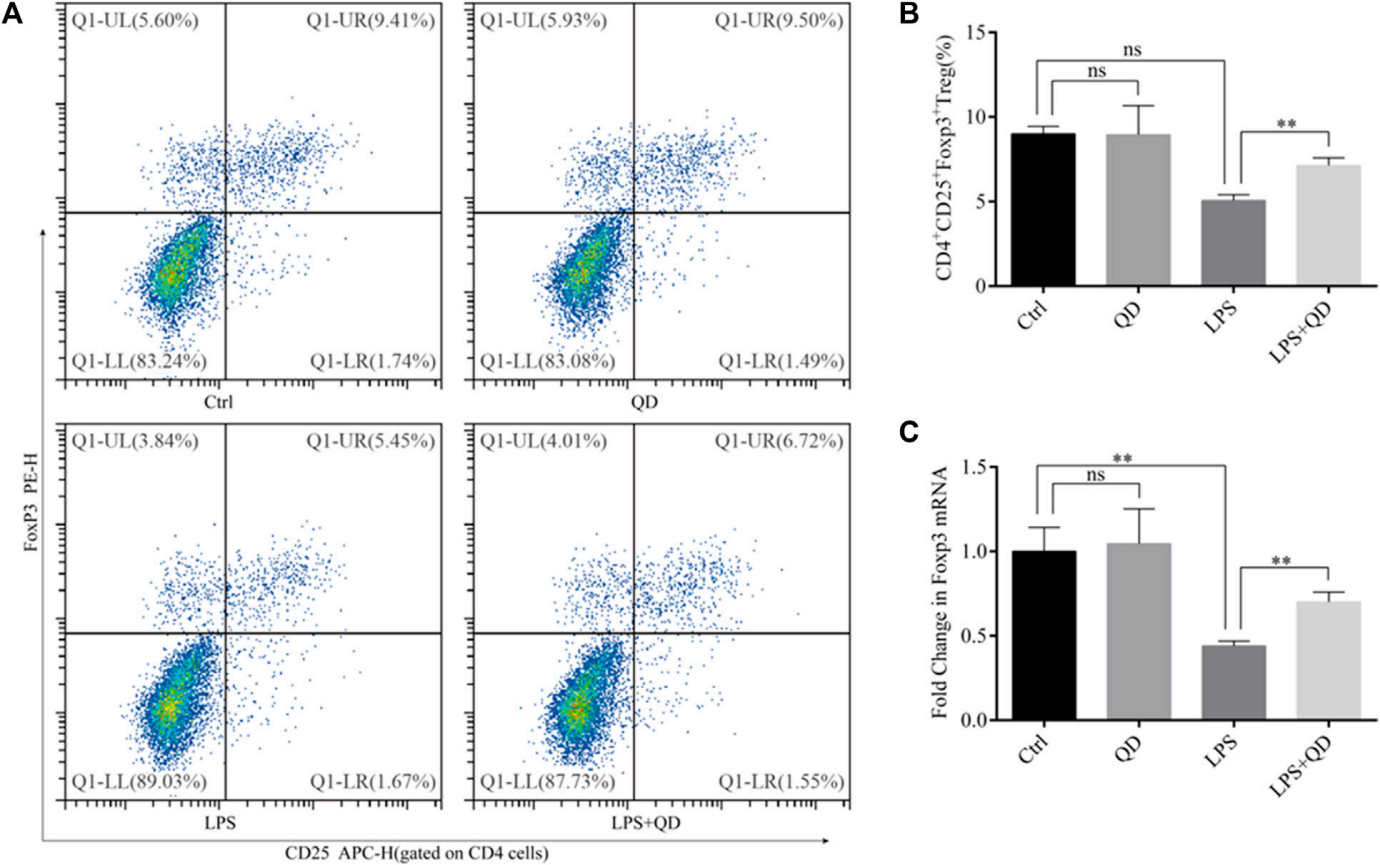
QD induces CD4^+^CD25^+^Foxp3^+^ Tregs in the ALI mouse model **(A)**. Analysis of CD4^+^CD25^+^Foxp3^+^ Tregs in the peripheral blood from control and ALI model mice treated with or without QD. **(B)**. Quantification of peripheral blood Tregs from six control and six ALI model mice treated with or without QD. **(C)**. mRNA levels of Foxp3 from whole blood extractions.

Since CD4^+^CD25^+^Foxp3^+^ Tregs play crucial roles in the TGF-β mediated regulation of immune response in acute lung injury (D'Alessio et al., 2009), we next analyzed the levels of CD4^+^CD25^+^Foxp3^+^ Tregs in the peripheral blood from sham, LPS-instilled, and LPS-instilled QD-treated mice. Our data showed that LPS instillation resulted in decline of CD4^+^CD25^+^Foxp3^+^ Tregs while QD treatment restored the portion of CD4^+^CD25^+^Foxp3^+^ Tregs in the peripheral blood (Figures 2A, B). In addition, we found that the mRNA level of Foxp3 in the peripheral blood was reduced significantly in LPS treated mice compared to sham mice, but the reduction was attenuated by QD treatment (Figure 2C), suggesting that Foxp3 was transcriptionally activated.

### QD inhibits HDAC activates to permit H3K9ac modification at Foxp3 locus

**FIGURE 3:**
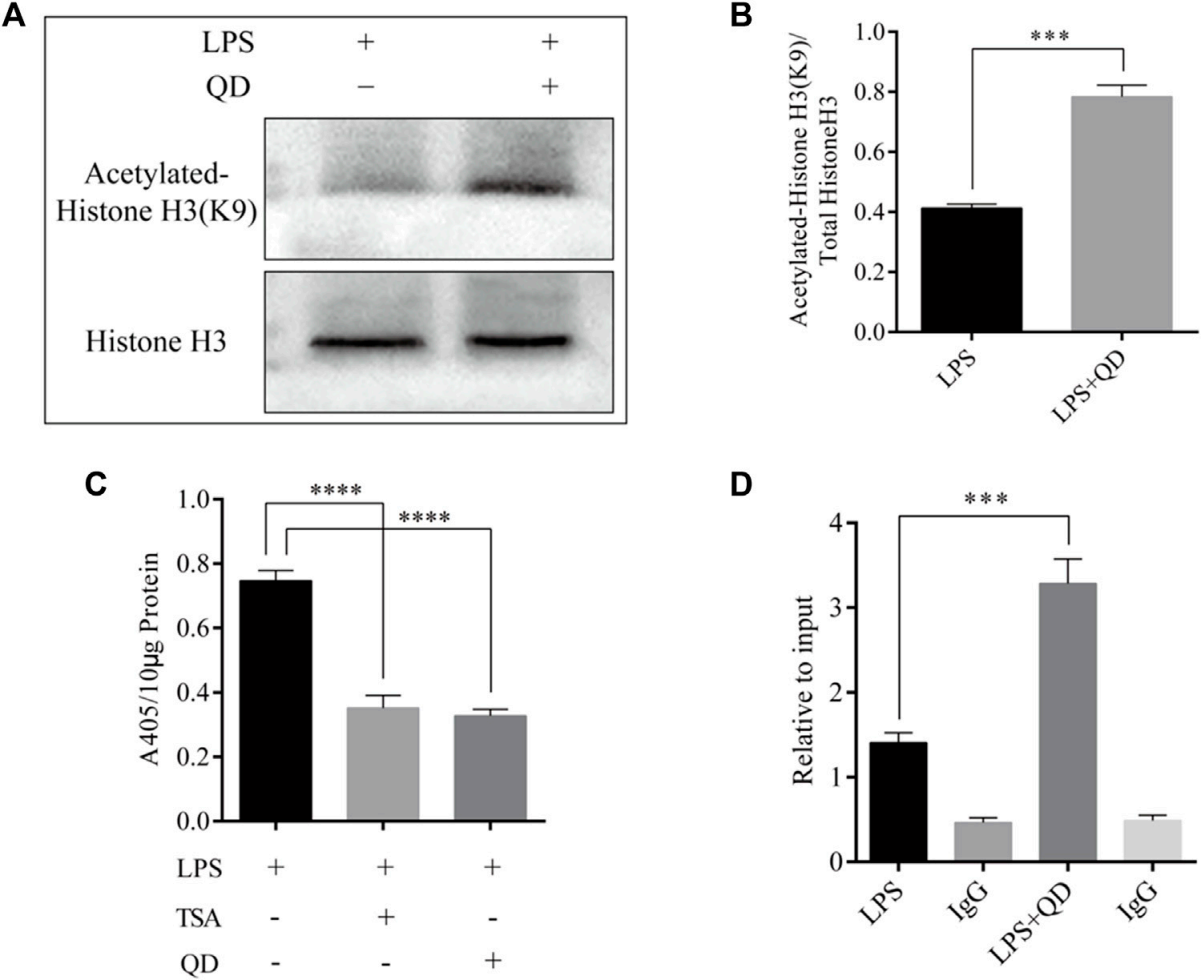
QD treatment leads to hyper acetylation of Histone H3 of the Foxp3 promoter in CD4^+^ cells **(A)**. ChIP-qPCR analysis of the Foxp3 promoter region pulled down by acetylated Histone H3 or IgG antibody in CD4^+^ cells treated with or without QD and pretreated with LPS. **(B)**. Western blot analysis of H3K9Ac in CD4^+^ cells treated with or without QD and pretreated with LPS. **(C)**. Quantification of H3K9Ac levels in CD4^+^ cells treated with or without QD and pretreated with LPS. **(D)**. Chromogenic assays of HDAC activity in CD4^+^ cells lysates. TSA stands for Trichostatin A, and is a histone deacetylase inhibitor.

**FIGURE 4:**
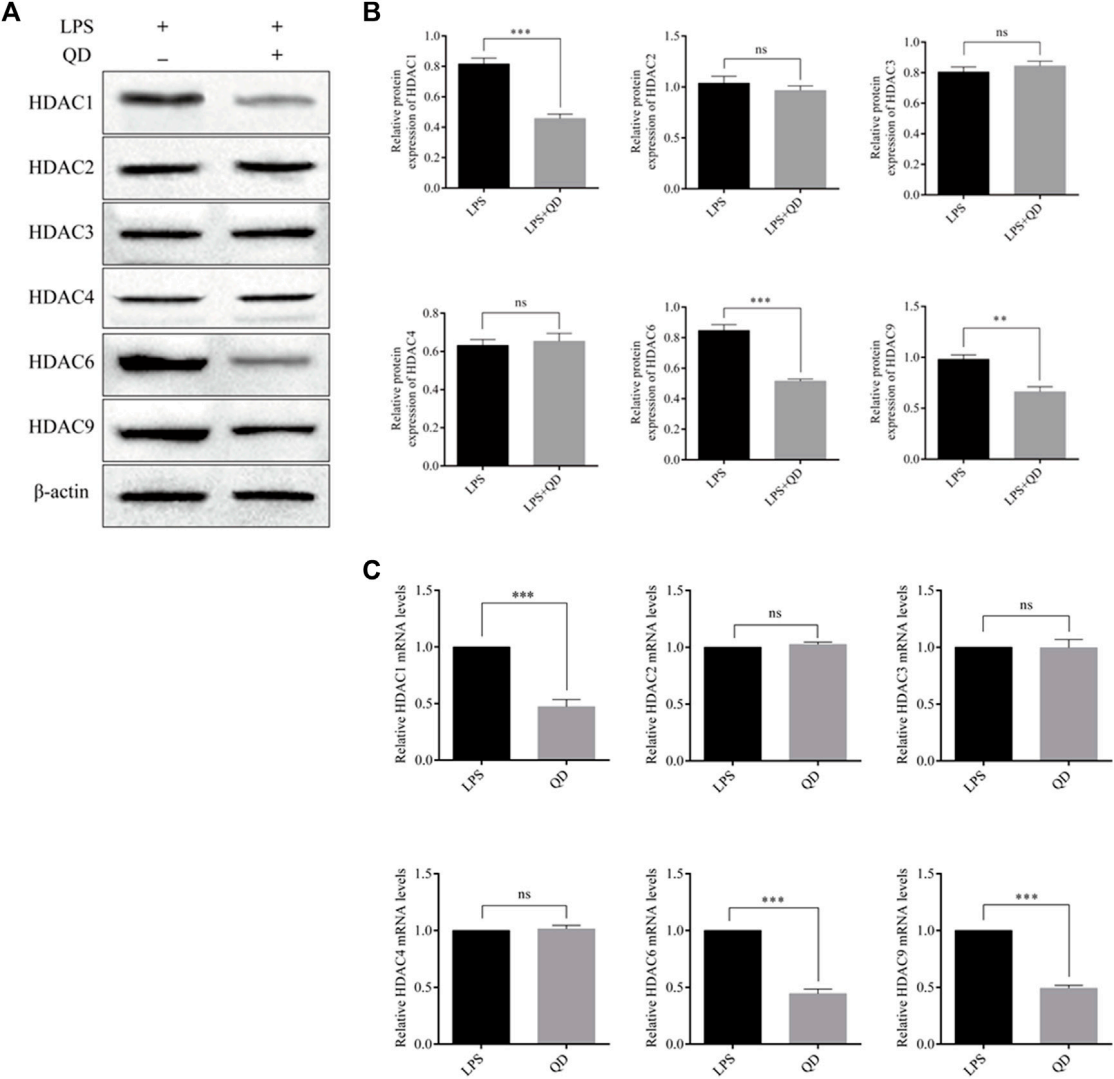
QD regulates HDACs in CD4^+^ cells **(A)**. Protein levels of HDACs in CD4^+^ cells treated with or without QD and pretreated with LPS. **(B)**. mRNA levels of HDACs in CD4^+^ cells treated with or without QD and pretreated with LPS.

To explore how Foxp3 was activated, we tested the effects QD has on epigenetic modification *in vitro* cultured CD4^+^ T cells and found that QD treatment dramatically increased overall acetylation of histone H3 on the ninth lysine residue (Figures 3A, B). Moreover, HDAC (histone deacetylase) activity was dramatically decreased after QD treatment (Figure 3C). We next utilized chromatin pull down assays to analyze the changes in H3K9Ac meditation in the promoter region of Foxp3 and found that OD treatment resulted in a distinct increase of H3K9Ac meditation in the promoter region of Foxp3 (Figure 3D). Hence, we analyzed the expression levels of HDACs *in vitro* cultured CD4^+^ T cells with or without QD treatment in the presence of LPS. Interestingly, the protein levels of HDAC1, HDAC6, and HDAC9 were all significantly decreased when cells were treated with QD (Figures 4A, B), as were the mRNA levels of HDAC1, HDAC6, and HDAC9 (Figure 4C). Taken together, our data suggested that QD inhibits the transcription of HADCs in CD4^+^ T cells which promotes the transcription of Foxp3 and facilitated the anti-inflammatory response.

### QD-treated macrophage facilitated Foxp3 activation in CD4^+^ T cells in response to LPS

**FIGURE 5:**
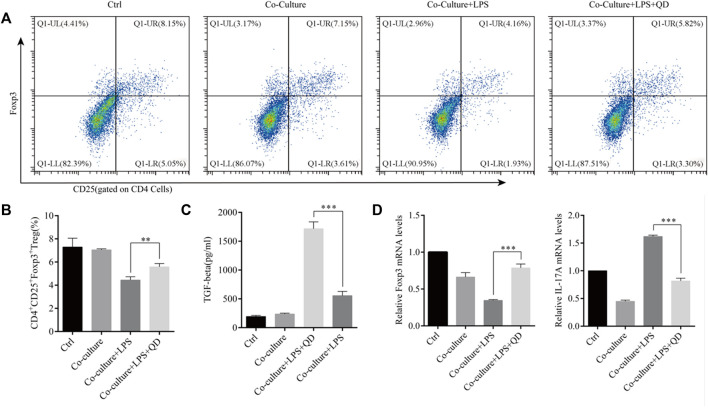
QD facilitates macrophage mediated Treg development Macrophages pretreated with or without QD were co-cultured with CD4^+^ cells treated with or without LPS. **(A)**. Analysis of CD4^+^CD25^+^Foxp3^+^ Tregs after 24 h co-culture. **(B)**. Quantification of CD4^+^CD25^+^Foxp3^+^ Tregs of co-cultured cells. **(C)**. The TGF-β level in the supernatant of co-cultured cells. **(D)**. qPCR analysis of Foxp3 and IL-17A from CD4^+^ cells in the co-culture.

To study the extrinsic effect QD has on CD4^+^ T cells, CD4^+^ T cells were co-cultured with macrophages pre-treated with or without QD. Interestingly, co-culture of macrophage with CD4^+^ T cells had limited effects on the activation of Foxp3, whereas co-culture of QD pre-treated macrophages with CD4^+^ T cells resulted in a significant increase the CD4^+^CD25^+^Foxp3^+^ Tregs proportion in response to LPS (Figures 5A, B), suggesting that QD-treated macrophage facilitated Foxp3 activation in CD4^+^ T cells in response to LPS. Importantly, we found that co-cultures of CD4^+^ T cells with QD pre-treated macrophage resulted in a prominent increase of TGF-β in the supernatant of co-culture medium in the presence of LPS (Figure 5C). Moreover, co-cultures of CD4^+^ T cells with QD pre-treated macrophages increased Foxp3 mRNA level but decreased the IL-17A mRNA level in response to LPS (Figure 5D).

### QD elevates β-catenin expression in macrophages to facilitate the development of CD4^+^CD25^+^Foxp3^+^ Tregs

**FIGURE 6:**
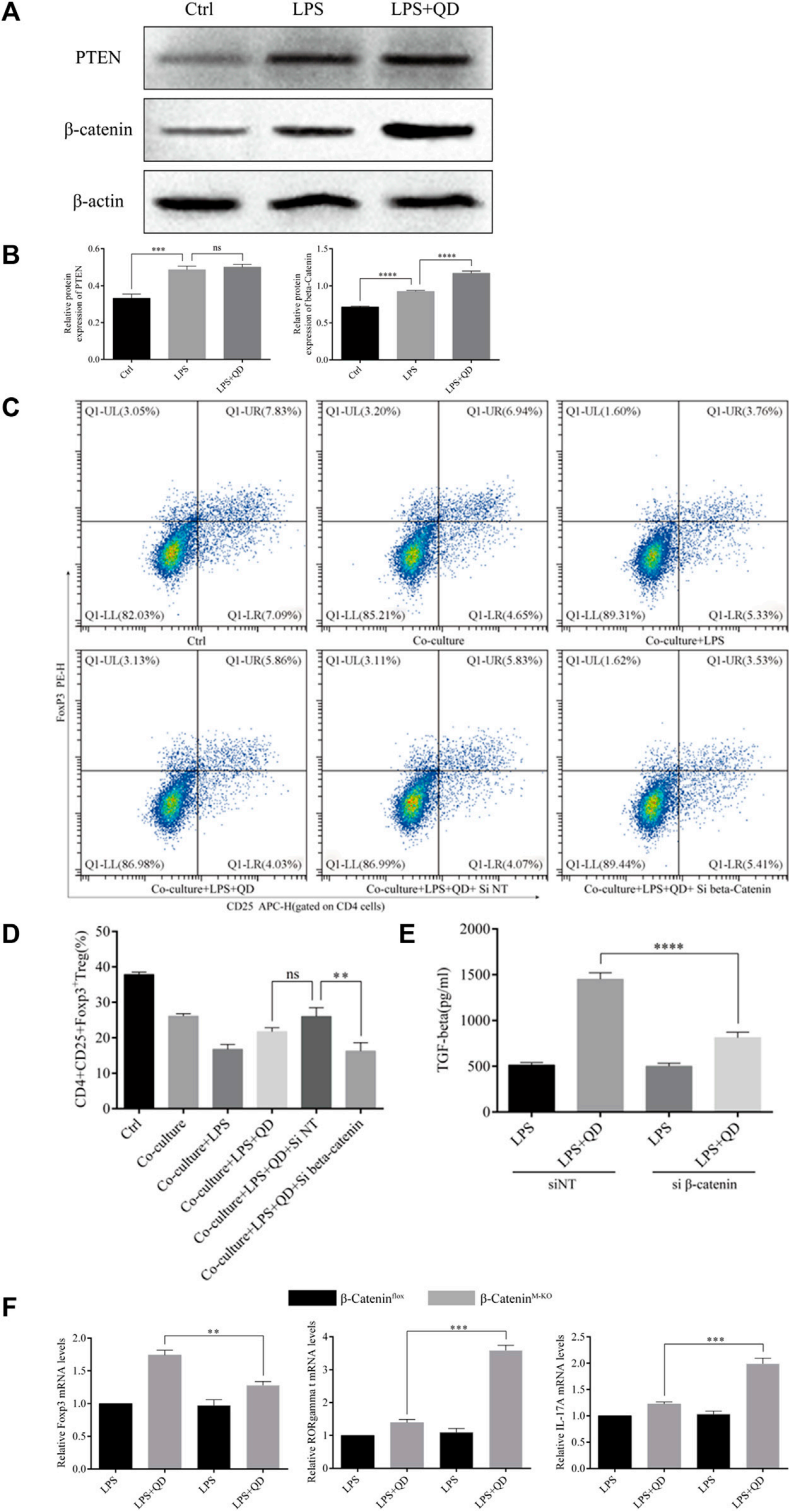
QD upregulates β-Catenin in macrophages **(A)**. Western blot analysis of PTEN and β-Catenin in macrophages treated with or without QD and pretreated with LPS. **(B)**. Quantification of protein levels of PTEN and β-Catenin in macrophages treated with or without QD and pretreated with LPS. Ctrl stands for control. **(C)**. Macrophages transfected with siRNA targeting β-Catenin were pretreated with or without QD and then co-cultured with CD4^+^ cells treated with or without LPS. **(D)**. Analysis of CD4^+^CD25^+^Foxp3^+^ Tregs after 24 h co-culture. **(E)**. The TGF-β level in the supernatant of co-cultured cells. **(F)**. qPCR analysis of Foxp3, RORγt, and IL-17A from CD4^+^ cells in the co-culture.

Since activation of PTEN and β-Catenin signaling in macrophages plays an essential role in the activation of Treg in acute lung injury (Zhou et al., 2019), we next examined the expression levels of PTEN and β-Catenin in macrophages treated with or without QD in the presence of LPS. Interestingly, the protein level of β-Catenin, but not PTEN, was further activated when LPS treated macrophages were further treated with QD (Figures 6A, B), suggesting that β-Catenin in macrophages mediated the Foxp3 activation. Hence, we inhibited the expression of β-Catenin in macrophages via siRNA and evaluated the role of β-Catenin in macrophage-mediated Foxp3 activation. Macrophages expressing a low level of β-Catenin failed to respond to QD and increased the proportion of CD4^+^CD25^+^Foxp3^+^ Tregs (Figures 6C, D). Moreover, the TGF-β level in the supernatant of co-culture medium decreased significantly when β-Catenin expression in macrophages was inhibited by siRNA (Figure 6E).

We next utilized myeloid β-Catenin deficient mice to study the role that macrophage-expressed β-Catenin has on T cells. We sorted CD4^+^ T cells from the co-culture with mouse macrophages with or without knockout of β-Catenin and analyzed the expression levels of Foxp3, RORγt, and IL-17A. As expected, macrophages expressing no β-Catenin failed to induce Foxp3 expression in CD4^+^ T cells but did induce RORγt and IL-17A expression in response to QD (Figure 6F). Taken together, these data indicated that QD induces β-Catenin expression in macrophages to facilitate the development of Tregs.

### Myeloid β-catenin is required for QD mediated CD4^+^CD25^+^Foxp3^+^ Tregs activation in LPS induced ALI mice model

**FIGURE 7:**
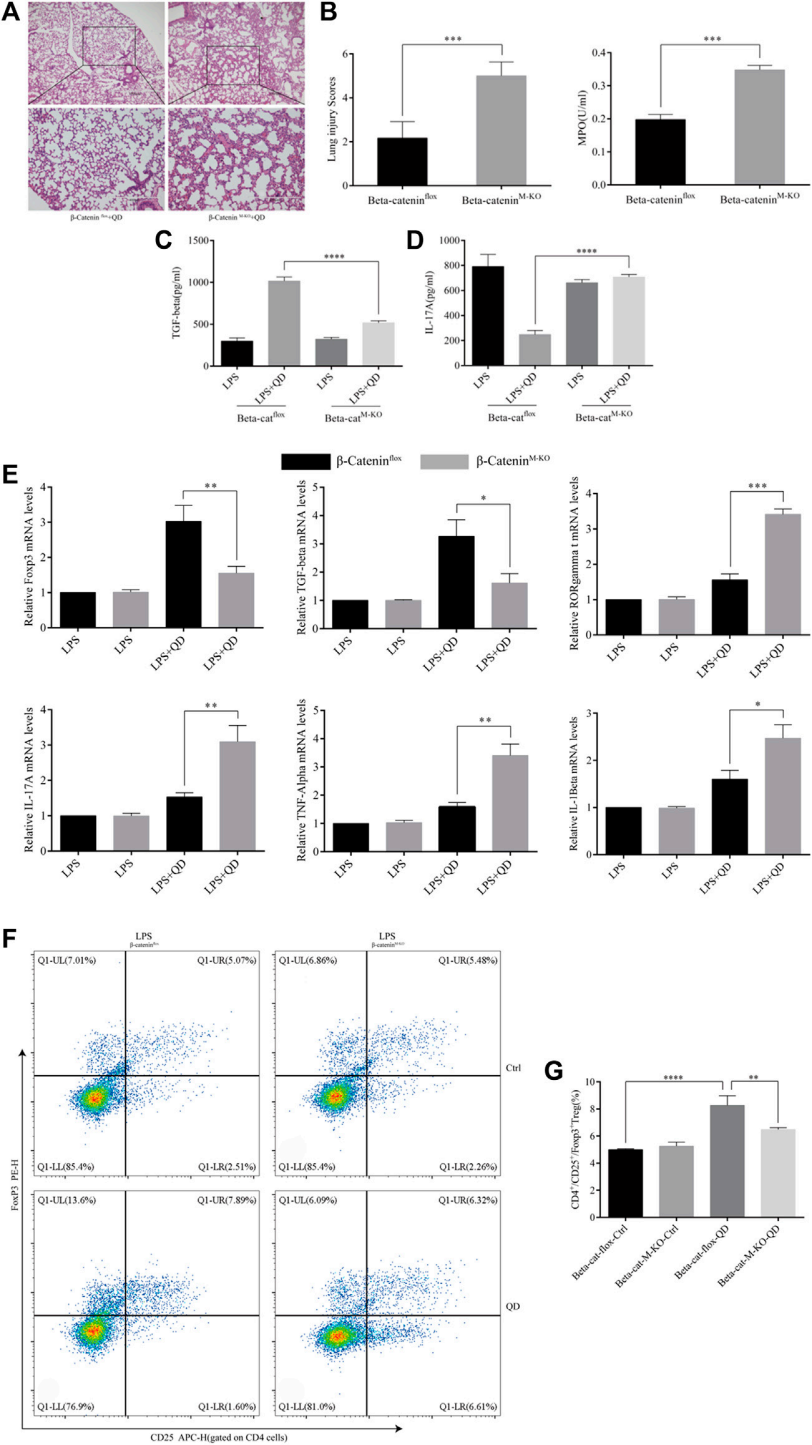
Myeloid β-Catenin is required for QD function **(A)** H&E staining of lung tissue from QD treated ALI model mice established from control or myeloid β-Catenin deficient mice (×40 magnification). **(B)**. Histopathological mean lung injury scores and myeloperoxidase (MPO) activity in lung homogenates of QD treated ALI model mice established from control or myeloid β-Catenin deficient mice. **(C)**. TGF-β levels in the serum from QD treated or untreated ALI model mice established from control or myeloid β-Catenin deficient mice. **(D)**. IL-17A levels in the serum from QD treated or untreated ALI model mice established from control or myeloid β-Catenin deficient mice. **(E)**. mRNA expression levels of Foxp3, TGF-β, RORγt, IL-17A, TNF-α, and IL-1β in the CD4^+^ cells from peripheral blood of QD treated or untreated ALI model mice established from control or myeloid β-Catenin deficient mice. **(F)**. Analysis of CD4^+^CD25^+^Foxp3^+^ Tregs in the peripheral blood from peripheral blood of QD treated or untreated ALI model mice established from control or myeloid β-Catenin deficient mice. **(G)**. Quantification of CD4^+^CD25^+^Foxp3^+^ Tregs in the peripheral blood from peripheral blood of QD treated or untreated ALI model mice established from control or myeloid β-Catenin deficient mice.

We next examined the curative effect that QD has on myeloid β-Catenin deficient mice *in vivo*. Mice failed to respond to QD treatment when β-Catenin was specifically knocked-out in myeloid cells (β-Catenin^M-KO^), as revealed by HE staining as well as lung injury scores (Figures 7A, B). Additionally, we monitored the myeloperoxidase (MPO) activity in lung homogenates from control ALI mice and myeloid β-Catenin deficient ALI mice and found that MPO activity increased in myeloid β-Catenin deficient ALI mice lung homogenates in comparison to wildtype ALI mice lung homogenates (Figure 7B). The TGF-β level decreased in the peripheral blood of myeloid β-Catenin deficient ALI mice treated with QD compared to wildtype ALI mice treated with QD (Figure 7C). However, IL-17A was induced in the peripheral blood of myeloid β-Catenin deficient ALI mice (Figure 7D). We isolated the lungs from myeloid β-Catenin deficient ALI mice and wildtype ALI mice and examined the mRNA expression levels of Foxp3, TGF-β, RORγt, IL-17A, TNF-α, and IL-1β. QD treatment failed to induce Foxp3 and TGF-β in myeloid β-Catenin deficient ALI mice lung but further induced pro-inflammatory factors such as RORγt, IL-17A, TNF-α, and IL-1β (Figure 7E). Finally, CD4^+^CD25^+^Foxp3^+^ Tregs in the peripheral blood of myeloid β-Catenin deficient ALI mice were significantly reduced in comparison to control ALI mice (Figures 7F, G). Hence, our data showed that β-Catenin in macrophages is required for QD mediated CD4^+^CD25^+^Foxp3^+^ Tregs activation in the LPS induced ALI mice model.

### QD inhibited GSK-3β mediated phosphorylation of β-catenin

**FIGURE 8:**
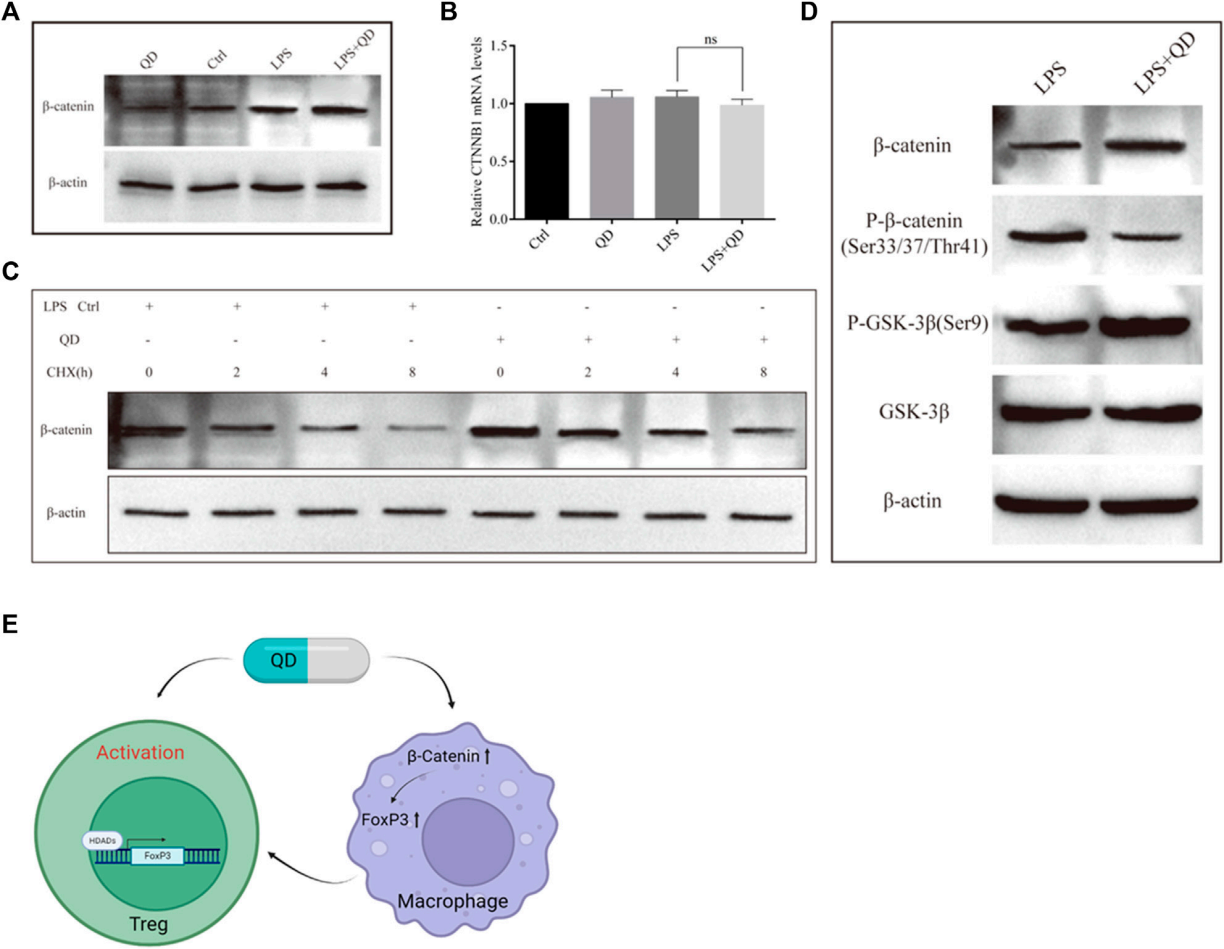
QD inhibits β-Catenin phosphorylation **(A)**. Western blot analysis of β-Catenin in macrophages treated with or without QD and pretreated with or without LPS. **(B)**. Quantification of protein level of β-Catenin in macrophages treated with or without QD and pretreated with or without LPS. **(C)**. Western blot analysis of β-Catenin in macrophages treated with CHX and pretreated with or without QD in the presence of LPS. **(D)**. Western blot analysis of β-Catenin phosphorylation in macrophages treated with or without QD in the presence of LPS. **(E)**. A diagram that illustrate the effects of QD has on Treg and macrophage which finally leads to the activation of Tregs and protect against LPS induced acute lung injury.

To understand how β-Catenin is activated in macrophages, we treated RAW264.7 cells with QD, LPS, or LPS together with QD. QD and LPS treatment increased β-Catenin protein level and treatment of LPS together with QD synergistically increased the β-Catenin protein level (Figure 8A). However, we found that none of the treatments altered the mRNA levels of β-Catenin (Figure 8B), suggesting that LPS and QD regulate β-Catenin post-transcriptionally. Hence, we treated the cell with CHX to inhibit overall protein synthesis and found that QD treatment postponed the degradation of β-Catenin (Figure 8C). Since phosphorylation of β-Catenin is crucial for β-Catenin stability, we examined the phosphorylation of β-Catenin with or without QD treatment in the presence of LPS. QD treatment caused reduced phosphorylation of β-Catenin at Ser33/37 and Thr41 sites via promoting phosphorylation of GSK-3β at Ser9 (Figure 7D).

## Discussion

As the master regulators of the immune system, CD4^+^CD25^+^Foxp3^+^ Tregs guard the guardians in cell-mediated immunopathology (Rogers and Williams, 2018). Tregs have long been proposed as beneficial regulators of ALI (D'Alessio et al., 2009). The differentiation of naïve CD4^+^ T cells to CD4^+^CD25^+^Foxp3^+^ Tregs is critical to reducing the damage of inflammation and hence reduceing the severity of ALI (Chai et al., 2020). Our data showed that QD acts intrinsically and extrinsically to promote the expression of Foxp3 in T cells to balance the immune environment and protect against ALI. QD treatment caused not only increase of TGF-β, but also decrease of IL-17A, IL-23 and TNF-α. We have shown that the percentage of Tregs in the peripheral blood was increased but we did not check other types of immune cells in LPS induced ALI in the presence of QD. Due to complexity of the decoction, QD may also affect the development of other types of immune cells to reach a balance that favors the host to fight against ALI. Notably, we found that the activation of Foxp3 mediated by QD was achieved by hyper-acetylation of the Foxp3 promoter. This is in line with previous evaluations of utilizing HDAC inhibitors to enhance Treg function post-transplantation (Akimova et al., 2010; Wang et al., 2018). Loss of HDAC3 and HDAC6 resulted in attenuated endothelial barrier dysfunction in ALI model (Joshi et al., 2015), while HDAC9 has been proven important in regulating Foxp3-dependent T cell development (Tao et al., 2007). Our data revealed that the levels of HDAC1, HDAC6, and HDAC9 were all decreased in response to QD treatment, suggesting QD is a broad-spectrum inhibitor of deacetylases. However, how QD decreased the mRNA levels of HDACs is ambiguous.

A previous study demonstrated that myeloid β-Catenin signaling is essential for the induction of CD4^+^CD25^+^Foxp3^+^ Tregs in response to lung injury (Zhou et al., 2019). Additionally, β-Catenin signaling has been shown to participate in tissue fibrosis after injury (Xiong et al., 2015; Qiao et al., 2018). We established a myeloid specific knock-out of β-Catenin and found that QD stabilizes β-Catenin in macrophages, which further promotes the differentiation of Tregs extrinsically. Interestingly, β-Catenin expression in macrophages is not only required for Foxp3 and TGF-β activation in the lung in response to QD treatment, but also to suppress the pro-inflammation cytokines in the lung, which indicates that macrophages receiving QD exhibit comprehensive regulatory functions in the immune environment. Our results showed that the stability rather than the transcription of β-Catenin was modulated by QD in macrophages while QD treatment affects the transcription of Foxp3 in T cells. These two fundamentally different regulatory mechanisms may arise from the complexity of QD itself. Moreover, since the acetylation of β-Catenin itself has a essential role in activating Wnt signaling (Chen et al., 2020b) and regulating inflammation (Li et al., 2019), it is of significance to further dissect the role of Wnt signaling in T cells in response to QD. Taken together, our data unveiled synergetic effects of QD on T cells and macrophages in protecting against ALI, providing detailed insights of how a TCM decoction balances innate immunity at cellular and molecular levels.

## Data Availability

The original contributions presented in the study are included in the article/Supplementary Materials, further inquiries can be directed to the corresponding author.
